# The Sigma Factor AsbI Is Required for the Expression of Acinetobactin Siderophore Transport Genes in *Aeromonas salmonicida*

**DOI:** 10.3390/ijms24119672

**Published:** 2023-06-02

**Authors:** Diego Rey-Varela, Miguel Balado, Manuel L. Lemos

**Affiliations:** Departmento de Microbiología y Parasitología, Instituto de Acuicultura, Universidade de Santiago de Compostela, 15782 Santiago de Compostela, Spain; diegoreyvarela@gmail.com (D.R.-V.); miguel.balado@usc.es (M.B.)

**Keywords:** *Aeromonas salmonicida*, siderophores, acinetobactin, sigma factors, regulation

## Abstract

*Aeromonas salmonicida* subsp. *salmonicida* (*A. salmonicida*), a Gram-negative bacterium causing furunculosis in fish, produces the siderophores acinetobactin and amonabactins in order to extract iron from its hosts. While the synthesis and transport of both systems is well understood, the regulation pathways and conditions necessary for the production of each one of these siderophores are not clear. The acinetobactin gene cluster carries a gene (*asbI*) encoding a putative sigma factor belonging to group 4 σ factors, or, the ExtraCytoplasmic Function (ECF) group. By generating a null *asbI* mutant, we demonstrate that AsbI is a key regulator that controls acinetobactin acquisition in *A. salmonicida*, since it directly regulates the expression of the outer membrane transporter gene and other genes necessary for Fe-acinetobactin transport. Furthermore, AsbI regulatory functions are interconnected with other iron-dependent regulators, such as the Fur protein, as well as with other sigma factors in a complex regulatory network.

## 1. Introduction

Iron is an essential nutrient for all living beings, including bacteria, but its solubility in physiological conditions is extremely low [1]. Therefore, in order to extract the necessary iron from their hosts, most pathogenic bacteria produce small metallophores, known as siderophores [2,3]. *Aeromonas salmonicida* subsp. *salmonicida* (hereafter *A. salmonicida*) is a Gram-negative γ-proteobacteria which is the causative agent of furunculosis, a devastating disease with high morbidity and mortality affecting salmonids and many other fish species of high commercial value, causing great economic losses in the aquaculture industry worldwide [4]. *A. salmonicida* possesses two siderophore systems that produce the catechol siderophores acinetobactin and four different amonabactins [5,6,7,8]. Acinetobactin was firstly described in the human pathogen *Acinetobacter baumannii* (*A. baumannii*) [9], while amonabactins were first identified in *Aeromonas hydrophila* (*A. hydrophila*) [10]. The main genes involved in synthesis and transport of both siderophores and the biosynthetic and uptake pathways were described by our group in previous studies [7,11,12]. 

The amonabactin genes are conserved in most *Aeromonas* species and, thus, are part of the *Aeromonas* core genome. By contrast, the acinetobactin siderophore system is present only in *A. salmonicida,* and the most feasible hypothesis is that it was acquired through horizontal gene transfer [7]. This hypothesis is supported by the fact that this gene cluster is homologous to the clusters of the siderophore pseudomonine of *Pseudomonas entomophila* [13] and to the acinetobactin cluster of *A. baumannii* [14]. These two siderophores differ only in the presence of a salicylic moiety in the catechol group of pseudomonine [7]. *A. salmonicida* clearly produces acinetobactin, as has been demonstrated by chemical characterization [7]. Thus, although most *A. salmonicida* strains could simultaneously produce both acinetobactin and amonabactins, some strains isolated from fish raised in seawater produce only acinetobactin due to a deletion present in the amonabactin biosynthetic key gene *amoG* [7]. Once synthetized, the siderophores are exported through bacterial membranes to the external media where they bind Fe^+3^, thus forming the ferric-siderophore complex, which is then internalized trough specific TonB-dependent transporters (TBDTs) [3]. Previous studies have demonstrated that *A. salmonicida* uses TBDTs for the internalization of siderophores, specifically the outer membrane transporter FstB for acinetobactin uptake [11] and FstC for amonabactins uptake [12].

Although iron is an essential nutrient, an excess of free forms of this metal can result in the formation of hydroxyl radicals that are toxic for bacterial cells. Thus, iron assimilation and storage systems are regulated by the intracellular concentration of Fe^+2^ [1,3]. In most bacteria, this regulation is mainly carried out through the Fur protein (Ferric uptake regulator), which is an iron-dependent transcriptional repressor that blocks the expression of genes related to iron uptake when internal levels of Fe^+2^ reach the required levels [15,16,17]. However, Fur is not the only regulator and most bacteria use other regulatory mechanisms, such as σ factors, to maintain the internal iron homeostasis [18].

The acinetobactin gene cluster of *A. salmonicida* encodes a putative RNA polymerase sigma-70 factor named AsbI, which belongs to the ECF subfamily, which has not been studied to date. These sigma factors are multi-domain subunits of bacterial RNA polymerase (RNAP) that play critical roles in transcription initiation, modulating the gene expression at the initiation of the transcription by modifying the affinity of the RNAP for DNA [19]. Therefore, in this work, we studied the role of RNAP sigma factor AsbI in the expression modulation of acinetobactin genes in *A. salmonicida*, analyzing the effect of *asbI* mutation in the activity of the promoters that control the expression of the acinetobactin siderophore system.

## 2. Results

### 2.1. Inactivation of asbI Reduces the Ability to Grow under Iron-Restricted Conditions 

**Figure 1:**
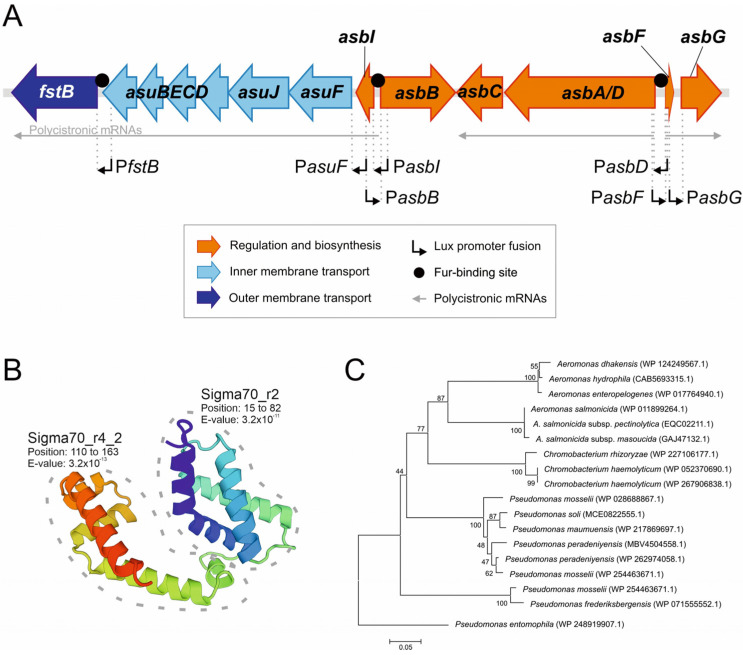
(**A**) Genetic map of the acinetobactin gene cluster in *A. salmonicida.* (**B**) Model of AsbI structure and Pfam conserved domain. The crystal structure of AlgU (6in7.1) was used as a template to build the model for AsbI. Pfam conserved domains analysis are denoted. (**C**) AsbI neighbor joining tree based on amino acid distances.

The putative ECF sigma factor AsbI (acc. No. ABO92290.1) is encoded within *A. salmonicida* amonabactin gene clusters (Figure 1A) and has a predicted structure composed by sigma domains 2 and 4 connected through a linker sequence (Figure 1B). This domain structure is typically found in ECF sigma factors, and is the minimum requirement for binding to its cognate promoter and RNA polymerase [20]. In addition, close homologues of AsbI (with more than 62% similarity) can be found in numerous *Aeromonas* and *Pseudomonas* species as part of other siderophore systems (Figure 1C).

**Figure 2:**
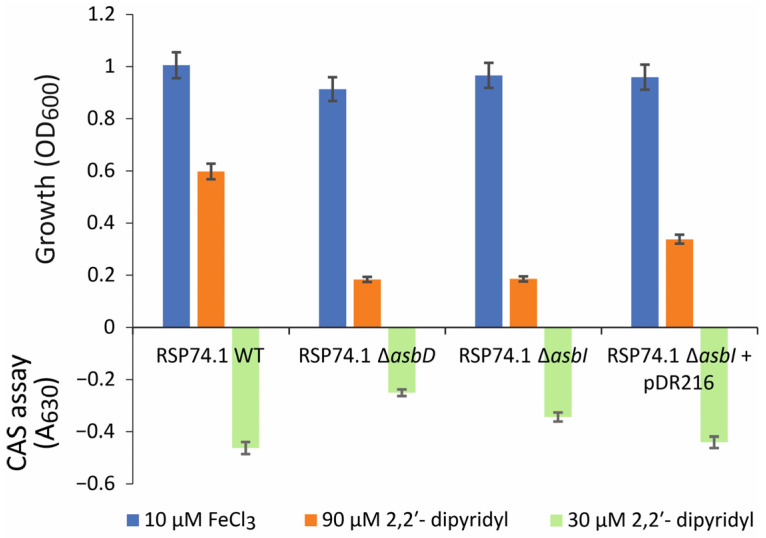
Growth (OD_600_) reached after 12 h of incubation of *A. salmonicida* RSP74.1 wild type, RSP74.1 Δ*asbD*, RSP74.1 Δ*asbI*, and RSP74.1 Δ*asbI* complemented with a copy of the wild *asbI* gene (pDR216) in CM9 minimal medium supplemented with FeCl_3_ (10 μM) or with the addition of the iron chelator 2,2′-dipyridyl (90 μM). Siderophore production was measured by the CAS assay at A_630_. The assay was performed in triplicate to calculate the standard deviation.

To study the role of AsbI, a deletion mutant of this gene was constructed in the *A. salmonicida* RSP74.1 background. This strain was isolated from a furunculosis outbreak in turbot and produces only acinetobactin, as it harbors a natural inactivation of the amonabactin biosynthesis genes [7]. Then, the ability of the RSP74.1 Δ*asbI* mutant to grow under low iron availability, and to produce acinetobactin, was compared to that of an RSP74.1 Δ*asbD* mutant, which is unable to produce any siderophore. All the strains assayed grew at the same level under iron-rich conditions (CM9 supplemented with FeCl_3_ 10 µM), achieving an OD_600_ of approximately 1 after 12 h of incubation (Figure 2). In contrast, since OD_600_ values ≤ 0.2 are attributed to the initial inoculum, mutant strains RSP74.1 Δ*asbI* and RSP74.1 Δ*asbD* were unable to grow in low iron availability (CM9 supplemented with the iron chelator 2,2′-dipyridyl at 90 µM). The parental strain RSP74.1 reached an OD_600_ value of 0.6 under this condition. The reintroduction by gene complementation of a functional copy of *asbI* in the mutant strain RSP74.1 Δ*asbI* partially restored its growth ability under low-iron conditions (Figure 2). This result strongly suggests that the inactivation of *asbI* reduces the ability of *A. salmonicida* to grow under low iron availability.

To study whether *asbI* is required to produce the siderophore acinetobactin, the strains were grown under weak iron-limiting conditions (CM9 with 2,2′-dipyridyl 30 µM) that do not limit the growth of any strain, and cell free supernatants were evaluated by the CAS assay in order to determine siderophore content. The CAS value found in the Δ*asbI* mutant supernatants was significantly higher than that found by the Δ*asbD* mutant (Figure 2), which suggest that the Δ*asbI* mutant still produces acinetobactin. Thus, the reduced ability to grow under low iron availability of this Δ*asbI* mutant would not be due to a lack of siderophore production.

### 2.2. AsbI Regulates the Expression of Acinetobactin Transport Functions, but Not Its Synthesis 

**Figure 3:**
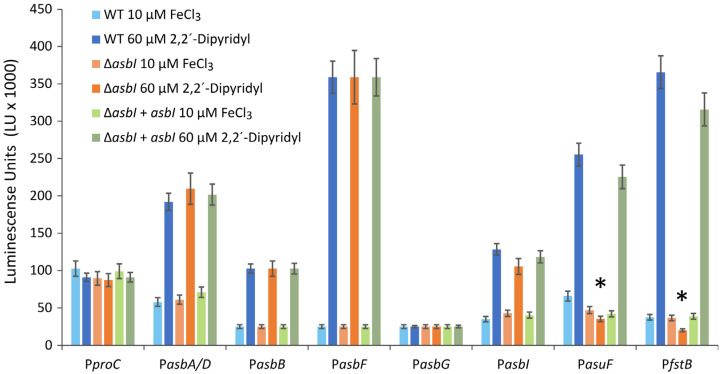
Transcriptional activity in the wild-type strain RSP74.1, RSP74.1 Δ*asbI,* and complemented strain RSP74.1 Δ*asbI* (pDR216 = pSEVA651-*asbI*) of the promoters that control the expression of acinetobactin main genes: P*asbD*, P*asbB,* P*asbF*, P*asbG*, P*asbI*, P*asuF,* and P*fstB*. Expression levels are showed in luminescence units. The constitutive promoter of the housekeeping gene *proC* (P*proC*) was used as reference. The luminescence activity was measured in CM9 minimal medium supplemented with FeCl_3_ 10 µM or with 2,2′-dipyridyl 60 µM. Asterisks denote statistically significant differences (Student’s *t*-test), * *p* < 0.05.

Since *asbI* would encode a putative transcriptional ECF sigma factor, we wanted to study whether it had a role in the modulation of acinetobactin gene expression. The transcriptional association of acinetobactin biosynthetic genes was previously studied, showing that *asbC* and *asbA/D* are cotranscribed in a polycistronic mRNA from a promoter upstream of *asbD*, and that *asbFG* could be expressed from a promoter located upstream of *asbG* (Figure 1A) [5]. In the present work, DNA regions immediately upstream of *asbG*, *asbF, asbD* and *asbB* (Figure 1A) were cloned into plasmid pSEVA236 upstream of a promoterless *lux* operon. Thus, the transcriptional activity of the promoters can be quantified as the luminescence produced by expression of the *lux* genes [21]. Resultant *lux* transcriptional fusions were named P*asbG*, P*asbF*, P*asbD*, and P*asbB*, respectively. Then, transcriptional fusions were conjugated into *A. salmonicida,* and luminescence was measured when the bacteria was cultured under high or low iron availability. The promoter of *proC* (P*proC* fusion) was used as a constitutive expression control [22,23]. The results of the assay are shown in Figure 3. 

All acinetobactin promoters significantly increase their transcriptional activity under low iron availability. By contrast, *lux* genes were constitutively expressed when transcribed under control of the *proC* promoter (P*proC*). The lowest transcription level was observed for promoter P*asbG*, and it did not show any response to iron concentration variations (Figure 3). Thus, DNA region upstream of *asbG* likely is not a real promoter and *asbFG* genes would be expressed together under control of the *asbF* promoter (P*asbF*). Under iron-rich conditions all acinetobactin promoters showed low activity (luminescence) and no differences were found between luminescence emitted by the fusions in the wild type and in *asbI* mutant (Figure 3). Interestingly, P*asuF* or P*fstB* promoters were almost non-luminescent under low-iron conditions in the Δ*asbI* mutant strain background. The mutant strain RSP74.1 Δ*asbI* complemented in trans with wild-type *asbI* (pDR216) showed the same expression pattern than the wild-type strain (Figure 3). These results clearly show that AsbI is required to express acinetobactin transport functions from promoters P*asuF* and P*fstB*. In addition, AsbI would not regulate its own expression. 

The acinetobactin transport genes are expressed as a polycistronic mRNA including *asuIFJDCEB-fstB* genes under the control of a promoter upstream of the *asbI* gene (P*asbI*) (Figure 1A) (Rey-Varela, Balado & Lemos, manuscript in preparation). Notably, Figure 3 shows that expression from other promoters upstream of *fstB* (P*fstB*) and *asuF* (P*asuF*) is preponderant, as they show higher transcriptional activity than P*asbI* under low-iron conditions. 

**Figure 4:**
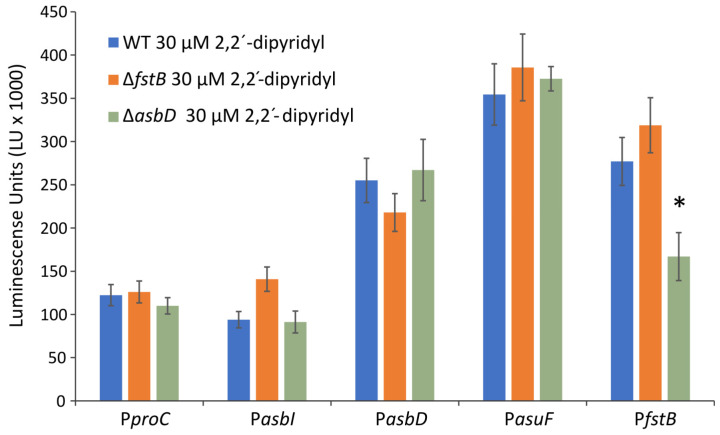
Transcriptional activity of the promoters P*asbI*, P*asbD*, P*asuF,* and P*fstB*, which control the expression of the main acinetobactin genes, in the wild-type strain RSP74.1, RSP74.1Δ*fstB* and RSP74.1Δ*asbD* mutants. Expression level is represented in luminescence units. The constitutive promoter of the gene *proC* (P*proC*) was used as reference. The activity was measured in CM9 minimal medium supplemented with 2,2′-dipyridyl 30 µM. Asterisk denote statistically significant differences (student’s *t*-test), * *p* < 0.05.

In other siderophore systems, the genes encoding transport functions increase their expression when the ferri-siderophore complexes are internalized [24]. To evaluate whether expression of the acinetobactin uptake genes in *A. salmonicida* respond to a ferri-acinetobactin presence, fusions with P*asbI*, P*asbD*, P*asuF*, and P*fstB* were introduced into the mutants RSP74.1 Δ*asbD* and RSP74.1 Δ*fstB*. The mutant Δ*fstB* produces acinetobactin but lacks the ferri-acinetobactin outer membrane transporter FstB, and it is therefore unable to use it as an iron source [11], while RSP74.1 Δ*asbD* does not produce acinetobactin. RSP74.1 Δ*fstB* showed expression patterns indistinguishable from the wild-type strain (Figure 4). This result strongly suggests that acinetobactin does not modulate the expression of its own transport genes. Nevertheless, RSP74.1 Δ*asbD* showed a significant lower expression for the promoter P*fstB* than for the wild-type strain, suggesting that this promoter requires the presence of acinetobactin for its expression.

### 2.3. Presence of Fur-Boxes in Acinetobactin Promoters

**Figure 5:**
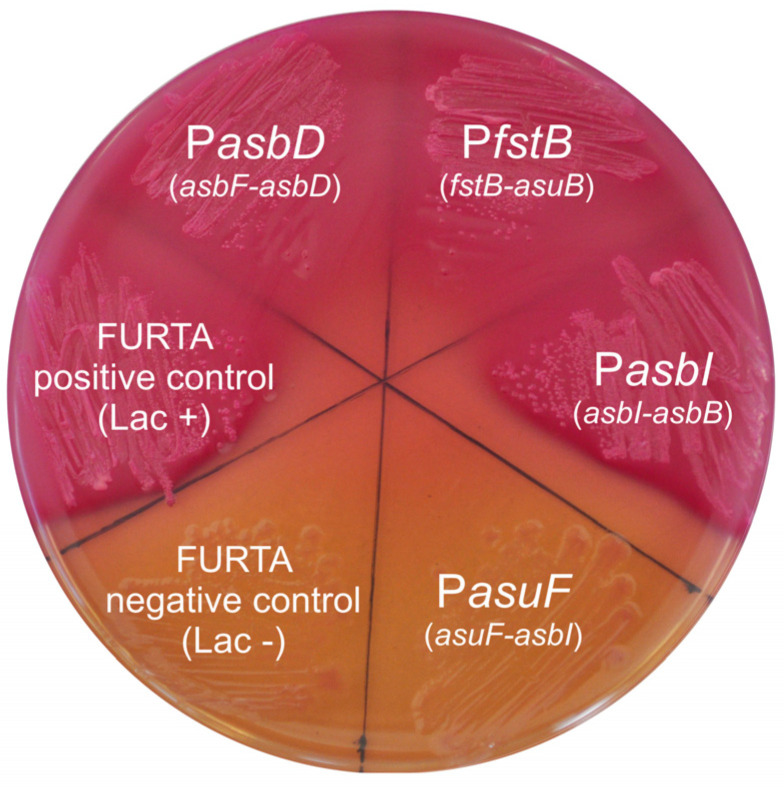
Fur titration assay (FURTA) for the promoters of intergenic regions of *fstB*, *asuF, asbI,* and *asbD*. Promoter regions were cloned into pT7-7 plasmid and introduced into *E. coli* H1717. Red-pink colonies indicate a Lac+ phenotype (bind *E. coli* Fur) while yellow-orange colonies indicate a Lac- phenotype (the introduced sequence does not carry a Fur box). Strain H1717 carrying an empty pT7-7 was used as FURTA negative control. Promoter of *Vibrio anguillarum fvtA* [25] was used as FURTA positive control.

Acinetobactin promoter regions contain a probable Fur box motif [5], which would mediate the strong iron response found in the expression pattern. To study whether acinetobactin gene promoters contain Fur box motifs, DNA regions of approximately 1 kb flanking *fstB-asuB*, *asuF-asbI*, *asbI-asbB*, and *asbF-asbD* intergenic sequences were tested in a Fur titration assay (FURTA) by using an *E. coli* H1717 indicator strain (Figure 5). 

*E. coli* H1717 harboring either *fstB-asuB*, *asbI-asbB*, or *asbF-asbD* sequences showed a FURTA positive phenotype (Lac+), which strongly suggests that Fur efficiently binds to them. By contrast, a FURTA-negative phenotype was observed when the *asuF-asbI* intergenic region was cloned in *E. coli* H1717 (Figure 5). This result suggests that P*asuF* transcriptional activity is independent of Fur. Thus, while P*fstB* is under the control of Fur and AsbI, P*asuF* would not be iron-regulated through the Fur repressor.

### 2.4. In Silico Prediction of AsbI Interactions with Other Proteins

**Figure 6:**
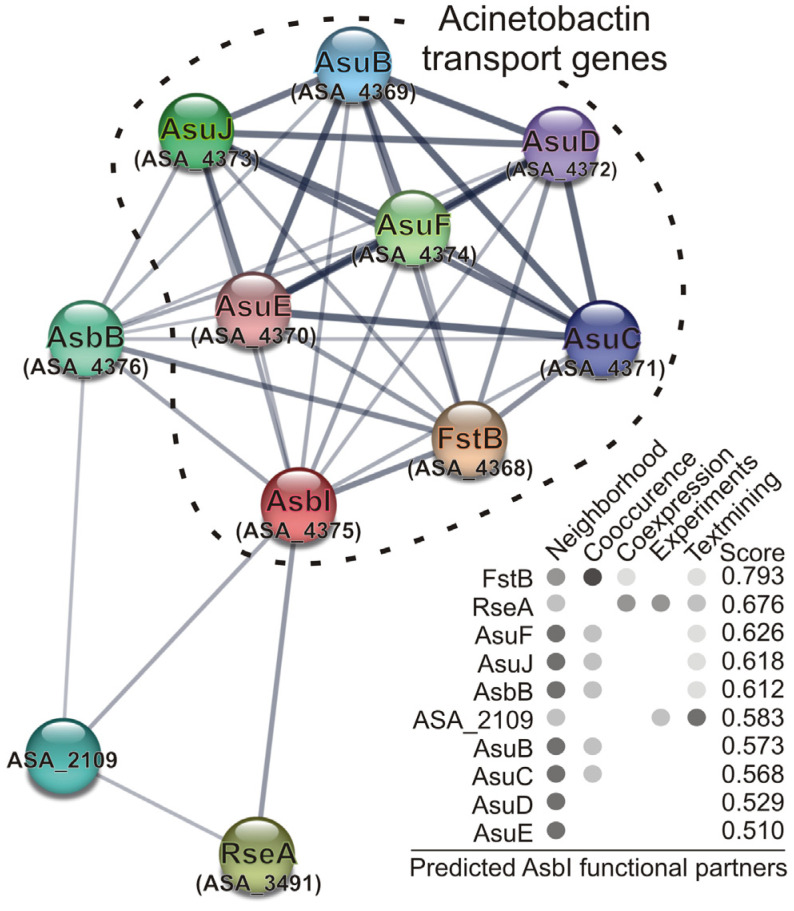
Prediction of protein–protein interactions, based on STRING Database v11.5 (https://string-db.org, accessed on 15 April 2023), of AsbI with other proteins involved in acinetobactin transport. The interactions include direct (physical) and indirect (functional) associations derived from the genomic context, high-throughput experiments, co-expression, and the literature. Colored nodes indicate query protein (AsbI) and first shell of interactors (limited to 10). Line thickness indicates the strength of data support. Loci codes refer to the genome sequence data of *A. salmonicida* subsp. *salmonicida* A449 (accession number CP000644.1).

Finally, prediction of protein–protein interactions based on the STRING Database (Figure 6) showed a strong association of AsbI with the acinetobactin transport functions, as well as strong interactions among them, which are mainly derived from their genomic context. This means that these proteins have more interactions among themselves than what would be expected for a random set of proteins of the same size and degree distribution drawn from the genome. Such an enrichment indicates that the proteins are at least partially biologically connected, as a group. It is noteworthy that there are also interactions with anti-sigma RseA (ABO91459.1) and locus ASA_2109 (ABO90175.1), encoding a putative two-component system hybrid sensor histidine kinase/response regulator. The role of these genes in the modulation of acinetobactin gene expression must be further studied.

## 3. Discussion

In this work, we describe the role of the putative sigma-factor AsbI in the expression of acinetobactin siderophore genes in the Gram-negative fish pathogen *A. salmonicida*. Usually, expression of most iron-acquisition-related genes is controlled by iron levels through the ferric-uptake regulator (Fur) [15,16,17]. However, additional control mechanisms have been described in many bacteria. One of these mechanisms involves the transcriptional regulators belonging to the Sigma (σ) factors family [26].

*A. salmonicida* AsbI belongs to group 4 σ factors, also known as the ExtraCytoplasmic Function (ECF) group, with roles in sensing and response to specific conditions that are generated outside of the cell or in the cell membrane [27]. The ECF group, with at least 43 distinct subgroups, is numerically the largest and most diverse group of sigma factors. Their roles include general stress response (*Methylobacterium extorquens* EcfG1), envelope stress response (*E. coli* σ^E^ or *Bacillus subtilis* σ^W^), oxidative stress (*Streptomyces coelicolor* σ^R^ and *Rhodobacter sphaeroides* σ^E^), or iron starvation [26]. The first described iron-related sigma factor was FecI from *E. coli,* which regulates the transcription initiation of the iron-citrate transporter genes *fecABCDE* [28]. Several studies have demonstrated the effects of these types of sigma factors on the iron metabolism, e.g., that pyoverdine biosynthetic genes in pseudomonads are under the control of the sigma factor PvdS [29]. More specifically, ECF sigma factors are usually involved in the regulation of the transport of siderophores, such as BupI and EcfI of *Bordetella bronchiseptica*, that control the expression of the siderophore receptors BfrZ and BfrH, respectively [30,31,32]. In *Pseudomonas aeruginosa*, FpvI mediates expression of *fpvA*, the pyoverdine transporter gene [33]. This is also the case for *A. salmonicida* AsbI reported here, which is required for the expression of genes that encode acinetobactin transporters located upstream of *fstB* and *asuF* genes. In addition, the FURTA assay results suggest that the promoter that controls expression of *fstB*, the acinetobactin TBDT transporter, is dually regulated through Fur repressors and AsbI, whereas the promoter of *asuF*, which controls the expression of the probable ABC efflux system, would be exclusively regulated by AsbI. This double regulation by Fur and ECF factors is present in other siderophore receptors. For instance, expression of BfrH, a putative siderophore receptor of *Bordetella bronchiseptica*, is regulated by Fur and by sigma factor EcfI [32]. 

The expression and activity of ECF factors can be controlled at several levels, but it is particularly prevalent the post-translational control by anti-factors that sequester these proteins, thus preventing their interaction with RNAP [26]. However, none of the genes adjacent to *asbI* or within the acinetobactin cluster of *A. salmonicida* are predicted to encode an anti-σ factor. Nevertheless, the STRING database showed an association of AsbI with anti-sigma RseA and a putative two-component hybrid sensor histidine kinase/response regulator of *A. salmonicida*. The relationship of these genes with the modulation of acinetobactin genes requires further investigation.

Some groups of ECF σ factors appear not to be controlled by any specific anti-σ factor and are directly regulated at the level of transcription [34]. The presence of a Fur-box upstream of *A. salmonicida asbI* indicates that, probably, it is directly controlled at the transcriptional level by the iron-dependent Fur protein. When iron is present, Fe(II) reversibly binds Fur, which facilitates the binding of Fur to its cognate sites (Fur-boxes), thus inactivating the expression of Fur-regulated genes [15,16,17]. In *Burkholderia cepacia* transcription of the OrbS ornibactin sigma regulator is controlled by Fur [35]. The transcription of biosynthetic genes of malleobactin in *Burkholderia pseudomallei* requires the sigma factor MbaS, and the *mbaS* promoter contains a Fur-binding site [36]. In *Pseudomonas aeruginosa*, a Fur-box was identified upstream of sigma-factor gene *fpvI*, consistent with the iron-regulated expression of this gene and its cognate pyoverdine transporter *fpvA* [33]. 

ECF sigma factors are often co-expressed with a second regulator which, in turn, transfers the extracellular signal to the cognate ECF sigma factor. For instance, *E. coli* FecR protein transmits the signal across the cytoplasmic membrane, through its transmembrane segment, into the cytoplasm, where it interacts through its short cytoplasmic portion with the FecI sigma factor which binds the promoter upstream of *fecA* gene [28,37]. In *Pseudomonas putida*, PupR is involved in the signal transduction for activation of PupI that regulates the synthesis of the outer membrane protein PupB [38]. *Bordetella bronchiseptica* HurR positively modulates HurI in the presence of heme to mediate the heme-dependent expression of BhuR, the outer membrane heme receptor [39]. Finally, in *Pseudomonas aeruginosa*, ferric pyoverdine induces expression of the outer membrane protein FpvA, the cytoplasmic membrane protein FpvR, and the two FecI-type sigma factors PvdS and FpvI in order to activate pyoverdine synthesis and uptake [24]. Bacterial sigma factors can be activated by their own siderophores produced by a bacterium or by xenosiderophores, iron-citrate, or heme groups [18,39]. In *A. salmonicida*, it seems likely that AsbI is regulated only by Fur, since our results demonstrate that neither acinetobactin nor acinetobactin transporter FstB are involved in *asbI* expression. Other global regulators, apart from Fur and ECF sigma factors, can regulate the expression of sigma factors involved in iron acquisition systems [40], such as AraC-type regulators such as PbtA of *V. anguillarum* [41].

In addition to the siderophore transport systems, iron-regulated ECF factors can control other functions related with iron acquisition during the infective process, such as proteases or hemolysins. For instance, the sigma factor PbrA is required for the transcription under low-iron conditions of the metalloprotease gene *aprA* in *Pseudomonas fluorescens* [42]. In *P. aeruginosa*, PvdS mediates the transcription of genes encoding pyoverdine biosynthesis but also the expression of other virulence factors such as exotoxin A, alkaline protease AprA, and one endoproteinase [24]. HurR positively modulates *hurP*, a gene encoding a prospective plasma membrane-associated protease in *Bordetella bronchiseptica* [39]. In accordance with this, it is likely that the regulator AsbI of *A. salmonicida* could regulate the expression of other virulence factors. Further works will be necessary in order to unravel these putative functions. 

In conclusion, we have demonstrated that the transport of the siderophore acinetobactin in *A. salmonicida* is regulated at the transcriptional level by the ECF sigma factor AsbI. This regulator is necessary for the expression of both the outer membrane Fe-acinetobactin transporter FstB and the putative acinetobactin exporter AsuF-J that is involved in acinetobactin secretion. Moreover, AsbI is probably regulated only by Fur and does not possess an anti-sigma factor counterpart. These findings increase our knowledge about the role of sigma factors in iron uptake mechanisms involving siderophores in bacteria. Additionally, since siderophores play a relevant role in bacterial virulence, external interference of these regulatory mechanisms could constitute a promising approach to combat bacterial infections [40].

## 4. Materials and Methods

### 4.1. Bacterial Strains, Plasmids and Culture Media

Strains and plasmids used in this work are listed in Table 1. *A. salmonicida* strains were cultured at 25 °C in Tryptic Soy Agar or Broth (Condalab, Madrid, Spain), supplemented with NaCl (Fisher Scientific, Barcelona, Spain) to a final concentration of 1% (TSA-1 or TSB-1, respectively). All *Escherichia coli* (*E. coli*) strains were cultured at 37 °C in Luria-Bertani medium (LB Lennox) (Condalab, Madrid, Spain). Culture media were supplemented with the suitable antibiotics when necessary. The antibiotics and final concentrations used were Kanamycin (Km; Merck KGaA, Darmstadt, Germany) at 50 μg mL^−1^; Chloramphenicol (Cm; Merck KGaA, Darmstadt, Germany), at 20 μg mL^−1^; and Gentamicin (Gm; Merck KGaA, Darmstadt, Germany), at 15 μg mL^−1^. The antibiotics were filter sterilized and stored at 4 °C. The growth under iron-limiting conditions was performed in M9 minimal medium supplemented with 0.2% Casamino Acids (BD-Difco, Madrid, Spain) (CM9) [43]. The iron-limiting conditions were induced by the addition of 2,2′-dipyridyl (Merck KGaA, Darmstadt, Germany) dissolved in milli-Q water at the appropriate concentration.

### 4.2. Mutant Construction and Complementation of asbI Gene

In-frame deletion mutants of the *asbI* gene were obtained by allelic exchange [47]. For the in-frame constructions, flanking regions of the gene were amplified by PCR and cloned into the pWKS30 plasmid (high copy number vector) [48]. The construction was sub-cloned into the suicidal vector pNidKan [46] into *E. coli* S17-1-λpir [49]. The strain carrying the construction was mated with *A. salmonicida* RSP74.1 (Cm^r^), and the exconjugants were selected with Cm and Km. The second recombinants were isolated from medium with sucrose (15%) and screened in Cm and Km plates. The Cm^r^ isolates were confirmed by a PCR for the target gene and subsequent DNA sequencing. For complementation of the Δ*asbI* mutant, the wild-type gene, together with its promoter, was amplified with Hi-Fidelity Kapa Taq (Merck KGaA, Darmstadt, Germany). The DNA fragment was cloned into the plasmid pSEVA651 [21] and mobilized into an *A. salmonicida* Δ*asbI* mutant from *E. coli* S17-1-λpir. All oligonucleotides used are listed in Table 2.

### 4.3. DNA Manipulation

Genomic DNA extraction was performed with the Easy-DNA kit standard protocol (Invitrogen, Fisher-Scientific, Barcelona, Spain) and the samples were stored at −20 °C until utilization. PCR reactions were carried out with *TaqK-*derived DNA polymerase NZY*Taq*II (NZYTech) in a thermocycler T-Gradient Thermal Cycler (Biometra, Göttingen, Germany). Plasmid extraction and purification were performed with the *GeneJET Plasmid Miniprep kit* (Thermo-Fisher). DNA bands from agarose gels and PCR were purified with NucleoSpin Gel and PCR Clean-up kit (Macherey-Nagel, Dueren, Germany).

### 4.4. Growth under Iron-Limiting Conditions

The ability to grow under iron-restricted conditions and the siderophore production of the parental and mutant strains were assayed in CM9 minimal medium [50] containing 2,2′-dipyridyl 90 µM as iron chelator. Bacteria were grown in TSB-1 for 5 h at 25 °C and cell concentration was adjusted to an optical density (OD_600_) of 0.5. This preinoculum was used to inoculate (1:40) CM9 medium in a 96-well microtiter plate (final volume of 200 µL per well). Plates were incubated for 18 h at 25 °C in an iMACK Microplate reader (Bio-Rad Laboratories, Madrid, Spain) taking OD_600_ measurements every 30 min. Media supplemented with FeCl_3_ 10 µM and non-inoculated media were used as controls. All conditions were carried out in triplicate within each plate and all experiments were repeated four times, calculating standard deviations and means for each condition. Student’s *t*-test was used for statistical analysis using SPSS software (version 20; IBM SPSS Inc., Chicago, IL, USA).

### 4.5. Quantification of Siderophores (CAS Assay)

Siderophore production was determined using the chrome azurol-S liquid assay (CAS) [51]. To induce siderophore production, bacteria were grown in CM9 medium containing 2,2′-dipyridyl 30 µM as iron chelator. Cell-free supernatants were obtained by centrifugation at 10,000 rpm in a Microcentrifuge (Eppendorf Ibérica, Madrid, Spain) and assayed for CAS reactivity as previously described [52]. Colorimetric reaction was measured in a spectrophotometer (Hitachi Europe, Buckinghamshire, UK) at 630 nm after 10 min of incubation. Non-inoculated medium was used as blank.

### 4.6. Promoter Fusions

DNA fragments which corresponded to the defined promoter regions and to the house-keeping gene *proC* were PCR amplified from genomic DNA and cloned into the reporter plasmid pSEVA236 [21]. The primers used are listed in Table 3. The promoters were cloned upstream of the *lux* genes into the MCS site in an *E. coli* S17-λpir strain. Since these genes lack a promoter, the transcription of *lux* genes is ruled by the promoter sequence cloned. The constructions were mobilized by conjugation into *A. salmonicida* RSP74.1 and its derivative mutants. The strains carrying the different promoter constructions were cultured under iron-rich and low-iron conditions (2,2′-dipyridyl 60 µM) in CM9 medium in microtiter plates in a final volume of 200 µL per well. The luminescence was recorded with a Tecan Infinite F200 reader (Tecan, Männedorf, Switzerland) after 16 h of incubation (OD_600_ ≈ 0.4). Statistical differences were determined using Student’s *t*-test using SPSS software (version 20; IBM SPSS Inc., Chicago, IL, USA). *p*-values were significant when *p* was <0.05.

### 4.7. Fur Titration Assay (FURTA)

Fur-regulated promoters were identified through a Fur titration assay (FURTA) [53]. The assay was performed as previously described [54]. Briefly, putative Fur-regulated promoters were cloned into the multicopy plasmid pT7-7 and transformed into *E. coli* H1717 [44] (strain kindly donated by Prof. Klaus Hantke from the University of Tübingen, Germany). This strain carries the Fur-regulated gene *fhuF*::*lacZ*, which is highly sensitive to changes in the concentration of the Fur repressor. The introduction in multicopy of a promoter that possesses a Fur-box causes the derepression of the *fhuF*::*lacZ* gene by binding the Fur protein, leading to the transcription of the *lacZ* gene. The expression of the LacZ protein leads to a Lac^+^ phenotype in the *E. coli* H1717 strain that can be visualized on MacConkey Agar plates supplemented with Fe_2_(SO_4_)_3_ 40 µM, ampicillin 100 μg mL^−1^ and 1% of extra lactose. The Lac+ phenotype is seen as red colonies in the medium, while the Lac- or FURTA negative is seen as yellow-orange colonies. The oligonucleotides used to clone the promoters into the plasmid pT7-7 were the same used for the transcriptional fusions (Table 3).

### 4.8. Bioinformatic Tools

STRING Database v11.5 (https://string-db.org/ accessed on 15 April 2023) was used to determine in silico, by means of the web tool, the protein–protein interactions between AsbI (ABO92290.1), and other *A. salmonicida* proteins derived from the genomic context, including direct (physical) and indirect (functional) associations, high-throughput experiments, co-expression, and the literature. The number of interactors represented was limited to 10 and a medium confidence interaction score (minimum of 0.4) was used.

## Figures and Tables

**Table 1:** Strains and plasmids used in this work.

Strain/Plasmids	Description	Reference
*Aeromonas salmonicida* subsp.* salmonicida*		
RSP74.1	Isolated from turbot, Portugal, Cm^r^	[5]
RSP74.1 Δ*asbD*	RSP74.1 defective in the acinetobactin synthesis gene *asbD*, Cm^r^	[5]
RSP74.1 Δ*fstB*	RSP74.1 defective in the acinetobactin transporter gene *fstB*, Cm^r^	[11]
RSP74.1 Δ*asbI*	RSP74.1 defective in the acinetobactin putative regulator gene *asbI*, Cm^r^	This study
*Escherichia coli*		
DH5α	Cloning strain	Laboratory stock
H1717	Strain used in FURTA assay *araD139* Δ*lacU169 rpsL150 relA1 flbB5301 deoC1 ptsF25 rbsR aroB fhuF::*λ placMu	[44]
Plasmids		
pT7-7	Cloning vector, Ap^r^	[45]
pNidKan	Plasmid with sucrose selection, Kan^r^	[46]
pDR216	pSEVA651 containing wild-type *asbI*	This study

**Table 2:** Oligonucleotides (Fisher-Scientific, Barcelona, Spain) used for the amplification of the flanking regions of *asbI* in *A. salmonicida* and subsequent cloning and mutation by allelic exchange.

Oligonucleotide	Sequence (5′ -> 3′)	Size (bp)
asbI_del_1_XbaI	CCGTCTAGATAAACGCCTTGAAGGCATCG	1018
asbI_del_2_BamHI	GGCGGATCCCTCAATGGTATGGGCTACCA	
asbI_del_3_BamHI	CCCGGATCCCCGATATAACGTCTGCCACT	905
asbI_del_4_XhoI	GCGCTCGAGTAAGCGTCATTTCAGGGCTG	
asbHJ_compl-1_XbaI	GCGTCTAGATTCCAGAGAAGGCAGATGCG	5132
asbJ_compl-2_BamHI	GCGGGATCCATCTGCTGCAATATGACCCC	

**Table 3:** Oligonucleotides (Fisher-Scientific, Barcelona, Spain) used for the amplification of the promoter regions in *A. salmonicida* and subsequent cloning into the pSEVA236 plasmid.

Oligonucleotide	Sequence (5′ -> 3′)	Size (bp)
Promoter fusion of *fstB*		
fstB_fusP-F-V2_KpnI	GCGGGTACCGCACAAACTCAAGCCGGATC	822
fstB_fusP-R-V2_XbaI	CCGTCTAGAAGGATGCAACAGATGGGGCT	
Promoter fusion of *asuF*		
asuF_fusP-F-V2_BamHI	GCGGGATCCGAGAGGGAAGCTTGCTCCTT	700
asuF_fusP-R-V2_XbaI	GCGTCTAGACGCGGCTTTGGAATCAAGAA	
Promoter fusion of *asbI*		
asbI_fusP-F-V2_BamHI	GCGGGATCCGGCACTCAATACCATGACGA	976
asbI_fusP-R-V2_XbaI	GCGTCTAGACTGTTCCTGCAAGGAGCAAG	
Promoter fusion of *asbD*		
asbD_fusPromot-1_BamHI	GCGGGATCCGCAGCAAATCGCATCACTCG	563
asbD_fusPromot-2_XbaI	GCGTCTAGAAACCTCACCCAGTGCTGAGT	
Promoter fusion of *asbB*		
asbB_fusP-F_BamHI	GCGGGATCCTGAGTGCAGGTTGCTGGGTT	551
asbB_fusPromot-2_XbaI	GCGTCTAGATTCATGAGTGCTCACAGGTA	
Promoter fusion of *asbG*		
asbG_fusP-F-V2_BamHI	GCGGGATCCAATGATTAGCCATCAATGGC	452
asbG_fusP-R-V2_XbaI	GCGTCTAGACCCGGCATCTTCTGATGACA	
Promoter fusion of *asbF*		
asbF_fusP-F_BamHI	GCGGGATCCTTTCAGCCACTGTCTGCATG	592
asbF_fusP-R-V2_XbaI	CCGTCTAGAAAGTTGCTGTACGCGCTCTT	
Promoter fusion of *proC*		
proC_fusP-R-V2_XbaI	GCGTCTAGAGATACTGCGGCTCATATTGC	800
proC_fusP-F-V2_BamHI	GCGGGATCCAGGAACGTATTGTACAGGCC	

## Data Availability

All DNA sequence data used in this work can be found at GenBank (NCBI), as part of the genome sequence data of *Aeromonas salmonicida* subsp. *salmonicida* A449, under accession number CP000644.1.
